# Heart Protection by Herb Formula BanXia BaiZhu TianMa Decoction in Spontaneously Hypertensive Rats

**DOI:** 10.1155/2019/5612929

**Published:** 2019-11-16

**Authors:** Jiaye Jiang, Dan Huang, Yuan Li, Zhongyuan Gan, Hanqing Li, Xindan Li, Ka Bian, Yan Ke

**Affiliations:** ^1^Teaching Experimental Center, Shanghai University of Traditional Chinese Medicine, 1200 Cailun Road, Shanghai 201203, China; ^2^School of Basic Medical Sciences, Shanghai University of Traditional Chinese Medicine, 1200 Cailun Rood, Shanghai 201203, China; ^3^Department of Biochemistry and Molecular Biology, George Washington University, Washington, DC 20052, USA

## Abstract

Modern research has shown that BanXia BaiZhu TianMa decoction (BBT) has the potential effect of lowering BP in vitro and in vivo. However, its therapeutic mechanism has not been clearly defined. The present study was designed to evaluate the protective effect of BBT on the heart by examining heart functioning and anti-inflammatory characteristics and to obtain scientific evidence for its further medical applications. BBT was extracted by decocting the herb extraction and analysed by HPLC. The left ventricular mass index (LVMI) was measured, and a histological examination of samples of the heart was performed. Inflammatory status was investigated by measuring tissue levels of interleukin-1 (IL-1), interleukin-6 (IL-6), tumour necrosis factor (TNF-*α*), inducible nitric oxide synthase (iNOS), and molecules of the nuclear factor κB (NF-κB) pathway. The BBT treatment significantly reversed the course of hypertension-derived heart damage. Meanwhile, the herb formula markedly reduced levels of IL-1, IL-6, TNF-*α*, and iNOS. In addition, the traditional compound suppressed the activity of the NF-κB pathway. The present study provides evidence of heart protection by BBT in SHRs. The action mechanisms may be partially attributable to the anti-inflammatory characteristic of the formula. Understanding the pharmacological action of BBT will benefit its impending use.

## 1. Introduction

Approximately 40% of adults aged 25 and above have been diagnosed with hypertension worldwide. The number of people with high blood pressure rose from 600 million in 1980 to 1 billion in 2008. Of these, complications from hypertension account for 9.4 million deaths worldwide every year [1]. In China, the prevalence of hypertension is approximately 35.533 million, and approximately 75 million American adults (32%) have high blood pressure—that is 1 in every 3 adults. Despite the vast amount of evidence on the benefits of antihypertensive medicines, hypertension is associated with higher incidences of organ damage, including myocardial hypertrophy and heart damage, and is still the leading risk factor for disease and death worldwide [2–4].

Increasing arterial blood pressure leads to organ damage via haemodynamic load, which directly results in hypertensive vasculopathy and eventually left ventricular hypertrophy. Nevertheless, other pathogenic factors that may be independent of pressure load have been identified as playing key roles in hypertensive end organ damage [5, 6]. It is noteworthy that the impact of risk factors applies especially to early-stage arterial hypertension, for which inflammation has been highlighted [7, 8]. Proinflammatory cytokines, such as interleukin-6 (IL-6), interleukin-1 (IL-1), tumour necrosis factor (TNF-*α*), and inducible nitric oxide synthase (iNOS), are critical to the onset of inflammation and are known to activate the nuclear factor NF-κB pathway [9]. Thus, therapeutic interventions to reduce activation of inflammatory cascade may prove beneficial for reducing end-organ damage and preventing the consequences of hypertension, including myocardial infarction and heart failure [10].

Traditional Chinese medicine (TCM) has been used widely in the treatment of the symptoms and signs underlying elevated blood pressure. BanXia BaiZhu TianMa decoction (BBT) is a preparation consisting of *Pinellia ternata* (*Pinellia ternata* Breit, Araceae; Origin: Gansu Province), *Rhizoma atractylodes macrocephalae* (*Atractylodes macrocephala* Koidz, Compositae; Origin: Zhejiang Province), *Rhizoma gastrodiae* (*Gastrodia elata* Bl, Orchidaceae; Origin: Yunnan Province), *Poria* (*Poria cocos* Wolf, Polyporaceae; Origin: Anhui Province), *Exocarpium citri grandis* (*Citrus grandis* Osbeck, Rutaceae; Origin: Ningxia Province), and *Radix et rhizoma glycyrrhizae* (*Glycyrrhiza uralensis* Fisch, Leguminosae; Origin: Gansu Province) and was first documented in the book Yi Xue Xin Wu (New Discovery of the Medicine) in the Qing dynasty. Its main effects are drying dampness, resolving phlegm, calming the liver, and quenching wind. It has been widely used to treat hypertension-related symptoms in clinical practice for centuries in China. The most common symptoms include headache, dizziness, nausea, and vomiting, which are characteristic of the liver-yang hyperactivity syndrome and fluid-retention syndrome. *Pinellia ternata* is used to dry dampness and dissipate phlegm, reduce adverse reactions, and stop vomiting, whereas Rhizoma gastrodiae is used to calm the liver, relieve wind, and stop dizziness, and *Atractylodes macrocephala* is used to dry the spleen and treat dampness. Recently, modern research has shown that BBT has the potential effect of lowering BP in vitro and in vivo. Its chemical composition, which includes gastrodia and guanosine, was previously determined [11]. Our previous studies revealed that BBT could improve endothelial function and myocardial hypertrophy in SHRs [12, 13]. In addition, the formula also reversed renal damage by reducing oxidative stress [14]. The current study aimed to investigate the effects of BBT on the progression of organ damage in spontaneously hypertensive rats (SHRs). Whether BBT attenuates inflammatory signalling was also evaluated using samples of heart from SHRs.

## 2. Materials and Methods

### 2.1. Reagents

Trizol was purchased from Invitrogen life technologies (USA), Real Master Mix and RT-PCR kit were purchased from Tiangen biochemical co., LTD (China), and NF-κB, p-NFκB, IκB, p-IκB, and p-IκK antibodies were purchased from Cell Signalling Technology (USA). TNF-*α*, IL-1*β*, and IL-6 antibodies were purchased from PEPROTECH (USA). Antimouse, antirabbit antibodies were purchased from Ming Rui Technology Co., LTD (China). Gastrodin reference substance (China), Guanosine reference substance (China), atractylenolide I reference substance (China), and atractylenolide III reference substance (China) were also acquired.

### 2.2. Preparation of BanXia BaiZhu TianMa Decoction (BBT)

BBT is composed of *Rhizoma gastrodiae*, *Rhizoma atractylodes macrocephalae*, *Rhizoma gastrodiae*, *Poria*, *Exocarpium citri grandis*, and *Radix et rhizoma glycyrrhizae*, with a ratio of 3 : 6:2 : 2:2 : 1, reaching a total weight of 100 g. The BBT was decocted twice with pure water (1 : 10 and then 1 : 5, w/v). The solution obtained was concentrated using a rotary vacuum evaporator (70°C–80°C) and dried in a vacuum oven, affording 29.34 g extract (yield: 29.34%).

### 2.3. HPLC Analysis

HPLC analysis was performed using Hewlett Packard Agilent 1100 series HPLC System, equipped with G1329A ALS Auto-sampler and G1315A Diode Array Detector (Agilent Technologies, USA). Sample solution was injected onto a Kromasil5-C18 column (250 mm × 4.6 mm, ID 5 *μ*m, Jiangsu Hanbon Science & Technology Co., Ltd., China), with the column temperature at 30°C. The mobile phase of gastrodin consisted of water : acetonitrile = 95 : 5. The gradient elution of guanosine was acetonitrile: water = 2%–5% for 0–25 min. Atractylenolide I and atractylenolide III were both methanol : water = 71 : 29. The flow rate was set at 1 mL/min, except for guanosine (0.6 mL/min), and the injection volume was 10 *μ*L. Detection was performed at UV 280 nm.

### 2.4. Animals and Drug Administration

All Wistar-Kyoto (WKY) rats and SHRs (male) 6 weeks of age (body weight 180 ± 10 g) were obtained from Shanghai Slack Laboratory Animal Co., Ltd. (Shanghai, China). All experimental procedures were conducted in accordance with the National Institutes of Health (NIH, USA) Guide for the Care and Use of Laboratory Animals and were approved by the ethics committee of Shanghai University of Traditional Chinese Medicine. The rats were allowed to acclimate to the environmental conditions for 1 week. They were housed in individual cages on a 12 h light-dark cycle in a room with the temperature of 24 ± 2°C and humidity control of 50%–60%, with ad libitum access to tap water and standard rodent chow. The animals were divided randomly into 4 groups (*n* = 12 for each group): (1) WKY group, (2) SHR group, (3) SHR + Captopril group (34 mg/kg), and (4) SHR + BBT group (8.64 g/kg). The rats were sacrificed on the 12th and 20th week, and blood was collected by the abdominal aortic method. Concurrently, the heart was carefully removed and sliced into two parts. The first part was snap-frozen in liquid nitrogen and kept at −80°C for protein and RNA extraction, and the second part was immersed in 10% neutral-buffered formalin for histopathological examinations.

### 2.5. Blood Pressure Measurement

Systolic blood pressure (SBP) was measured every 2 weeks using tail-cuff plethysmography (Shanghai Alcott Biotech Co., Ltd) as described previously [15].

### 2.6. Left Ventricular Mass Index (LVMI) Measurement

Before the end of the experiment, rats were kept in metabolic cages for 24 h to collect urine samples. Left ventricular mass index (LVMI) was measured as described previously [16].

### 2.7. Histopathology Examination

After the rats were sacrificed, the heart was quickly removed and cut into 4-*μ*m sections. The paraffin-embedded heart slices were finally deparaffinized, rehydrated, and stained with haematoxylin/eosin (HE) for microscopic study to assess changes in histology. The obtained preparations were visualized using light microscopy at a magnification of 200x. Afterwards, a pathologist colleague was asked to examine each section in at least 10 randomly selected nonoverlapping fields under a light microscope in a blind manner. Quantitative evaluation of the heart injury was performed according to a pathological scoring system (neutrophil infiltration, haemorrhage, and necrosis), ranging from 0 (normal) to 5 (severe), as previously described [17].

### 2.8. Quantitative Real-Time PCR

Total RNA was extracted from frozen heart samples using a Trizol one-step RNA isolation kit (Invitrogen, USA) following the manufacturer's protocol. Briefly, 2.5 *μ*g of total RNA was reverse-transcribed using a RT first strand cDNA synthesis kit (Tiangen biochemical co., LTD, Beijing, China), and synthesized complementary DNA was amplified by a standard PCR protocol using SYBR green PCR master mix (Tiangen biochemical co., LTD, Beijing, China). Primers were synthesized from Sangon Biotech Co. Ltd. (Shanghai, China). The sequences of rat-specific primers for TNF-*α*, IL-1, IL-6, iNOS, and GAPDH used in the study are listed in Table 1. Cycling conditions were as follows: 15 min preincubation at 95°C, 10 sec denaturation at 95°C, and 31 sec annealing at 58°C for 40 cycles using an ABI PRISM 7300 sequence detection system (Applied Biosystems). Each reaction was amplified in triplicate, and the threshold cycles (Ct) were calculated using the △△Ct method. Relative gene expression was normalized with GAPDH as an internal reference.

### 2.9. Protein Extraction and Western Blot

The heart tissue was immersed in ice-cold lysis buffer (50 mM Tris at PH of 7.5, 1 mM EDTA, 150 mM NaCl, 20 mM NaF, 0.5% NP-40, 10% glycerol, 1% protease inhibitor cocktail and 1% phosphatase inhibitor) homogenized on ice, and centrifuged at 12,000 rpm for 10 min at 4⁰C, and the supernatant was obtained. Equal amounts of protein (80 *μ*g) were separated by 10% SDS polyacrylamide gel electrophoresis (SDS-PAGE) and transferred to nitrocellulose membranes (Bio-Rad Laboratories, Hercules, CA, USA). After blocking, the membranes were incubated with primary antibodies at 4°C overnight. Antibodies used in the present study included: TNF-*α* (1204M073Rb, PEPROTECH.INC, 1 : 1000 dilution), NF-κB p65 (3034, Cell Signalling, 1 : 1000 dilution), pNF-κB p65 (3033S, Cell Signalling, 1 : 1000 dilution), IκB*α* (4812S, Cell Signalling, 1 : 1000 dilution), pIκB*α* (2697S, Cell Signalling, 1 : 1000 dilution), pIκK (2697P, Cell Signalling, 1 : 1000 dilution), iNOS (ab9485, Abcam, 1 : 500 dilution), IL-1*β* (1 : 1000 dilution), IL-6 (1204M086R6, PEPROTECH.INC, 1 : 1000 dilution), and GAPDH (ab8245, Abcam, 1 : 5000 dilution). After three washes with PBS-T buffer, the membranes were incubated with HRP-labelled goat antirabbit or -mouse IgG for 2 h at room temperature. The protein bands were detected using ECL reagents. Chemiluminescent signals were detected and analysed using the ChemiDoc XRS Imaging System (Tanon, Shanghai, China).

### 2.10. Statistical Analysis

All data are expressed as the mean ± SEM, and *n* refers to the number of animals. Multiple group comparisons were performed using one-way ANOVA and LSD tests with SPSS18.0. The differences were considered statistically significant when *P* < 0.05.

## 3. Results

### 3.1. HPLC Profiles of BBT

**Figure 1:**
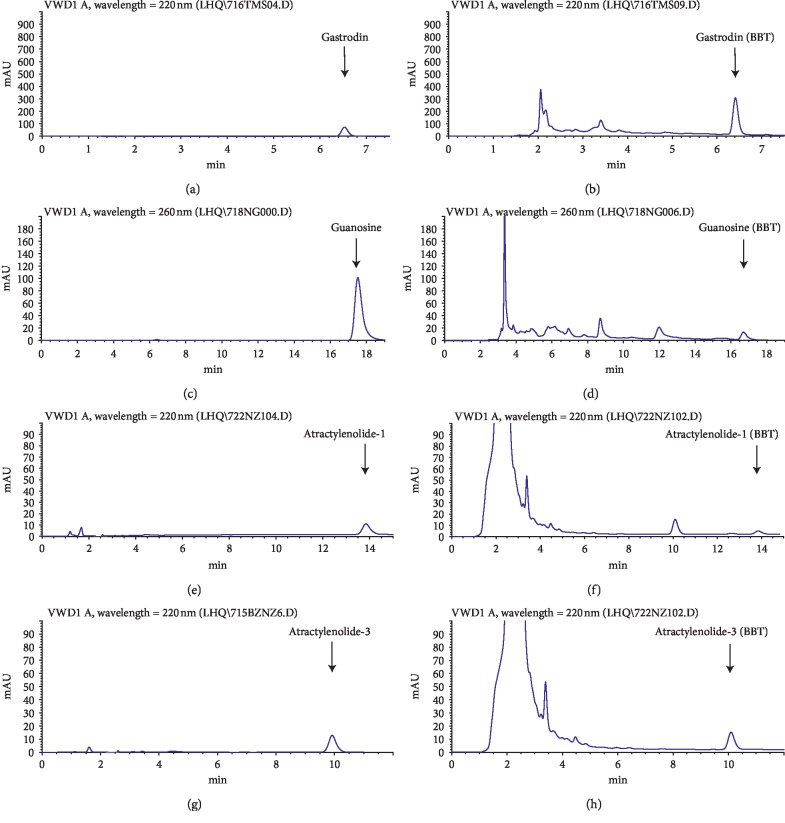
The typical HPLC chromatograms of BBT. Gastrodin (0.503%), guanosine (0.02%), atractylenolide-1 (0.014%), and atractylenolide-3 (0.027%) in BBT were determined.

The major components in BBT were analysed with HPLC (Figure 1). By comparison with the standard compounds, the main components in BBT were revealed to be gastrodin, guanosine, atractylenolide I, and atractylenolide III, with respective dosages of 0.503%, 0.02%, 0.014%, and 0.027%.

### 3.2. Effects of BBT on Blood Pressure

**Figure 2:**
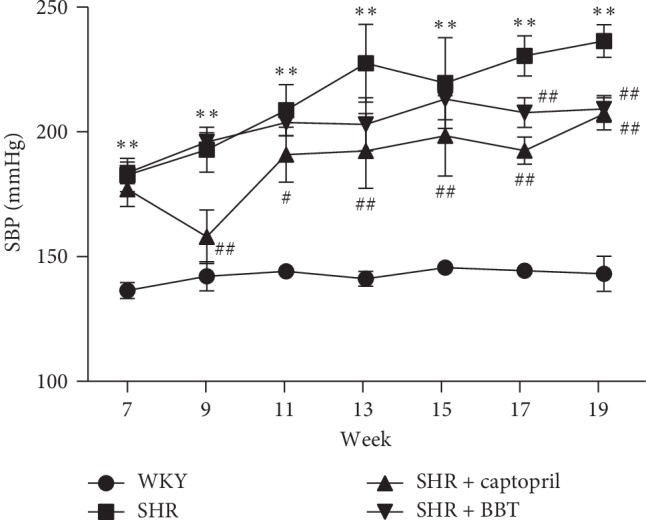
Time course of systolic blood pressure of the four groups of rats measured by tail-cuff method. Data are expressed as the mean ± SEM (*n* = 6 in each group). ^*∗∗*^*P* < 0.01 vs. WKY; ^#^*P* < 0.05 and ^##^*P* < 0.01 vs. SHR.

Systolic blood pressure, measured by tail-cuff method, was significantly increased at 7 weeks of age in the SHR group compared with the WKY group. However, systolic blood pressure in the BBT group was significantly decreased at week 17 compared with the SHR group (Figure 2).

### 3.3. Effects of BBT on Heart Function

Table 2 shows the markers of heart function in the four groups. The heart function markers, including LVMI, were significantly decreased at week 20 in the BBT-treated SHR group. However, the effect was not obvious at week 12 (Table 2).

### 3.4. Effects of BBT on Histopathological Examination

**Figure 3:**
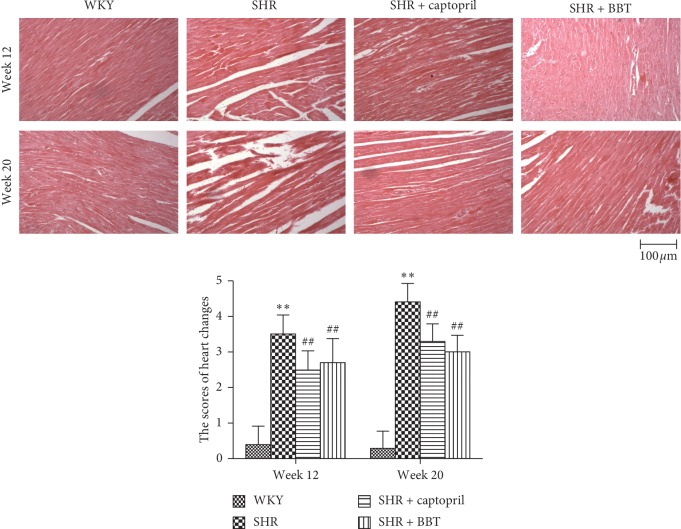
Effect of BBT on the heart histology (200 x). WKY at week 12 and week 20 showed no abnormal structure in the heart; SHRs showed glomerular capillary atrophy at week 12 and became more sever at week 20 in the heart; SHRs showed mild change at week 12 but showed that the sizes and gaps of myocardial cell significantly increased at week 20 in the heart; BBT at week 12 and week 20 showed the improvement effect both in the heart.

As shown in Figure 3, histopathological examination showed no obvious abnormality in rats' heart structures in the WKY group. However, at week 12, SHRs showed that necrosis obviously increased, which was aggravated at week 20. The heart-change scores were 0.40 ± 0.52 and 0.30 ± 0.48 in the WKY group at weeks 12 and 20, respectively. The heart-change scores were 3.50 ± 0.53 and 4.40 ± 0.52 in the SHR group at weeks 12 and 20, respectively (*P* < 0.01, *P* < 0.01). However, these pathological changes were partially prevented by BBT treatment at weeks 12 and 20 (*P* < 0.01, *P* < 0.01) (Figure 3).

### 3.5. Effect of BBT on the Expression of Inflammatory Factors

**Figure 4:**
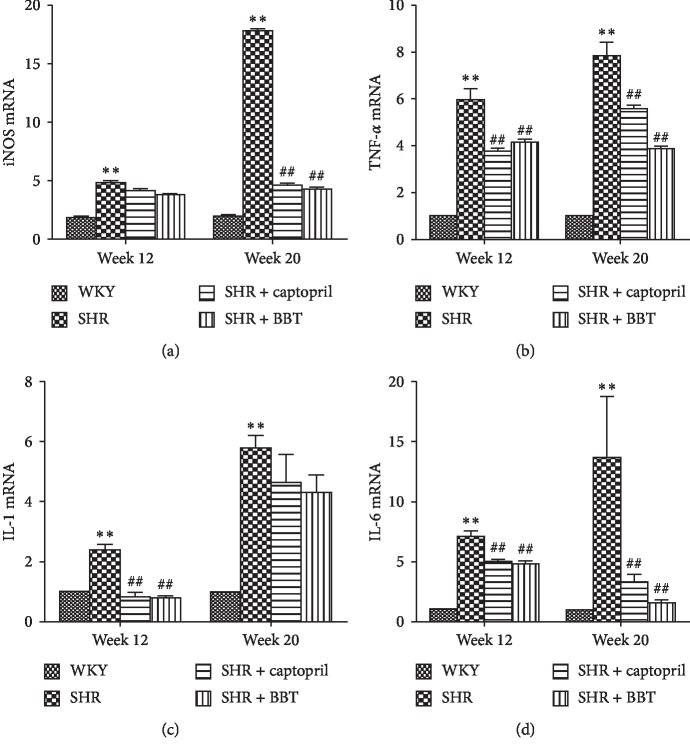
Effect of BBT on the mRNA expressions of inflammatory factors TNF-*α*, IL-1, IL-6, and iNOS in the heart. Data were obtained by real-time PCR, and the results were expressed as mean ± SEM relative to GAPDH (*n* = 6 in each group). ^*∗*^*P* < 0.05 and ^*∗∗*^*P* < 0.01 vs. WKY; ^#^*P* < 0.05 and ^##^*P* < 0.01 vs. SHR.

**Figure 5:**
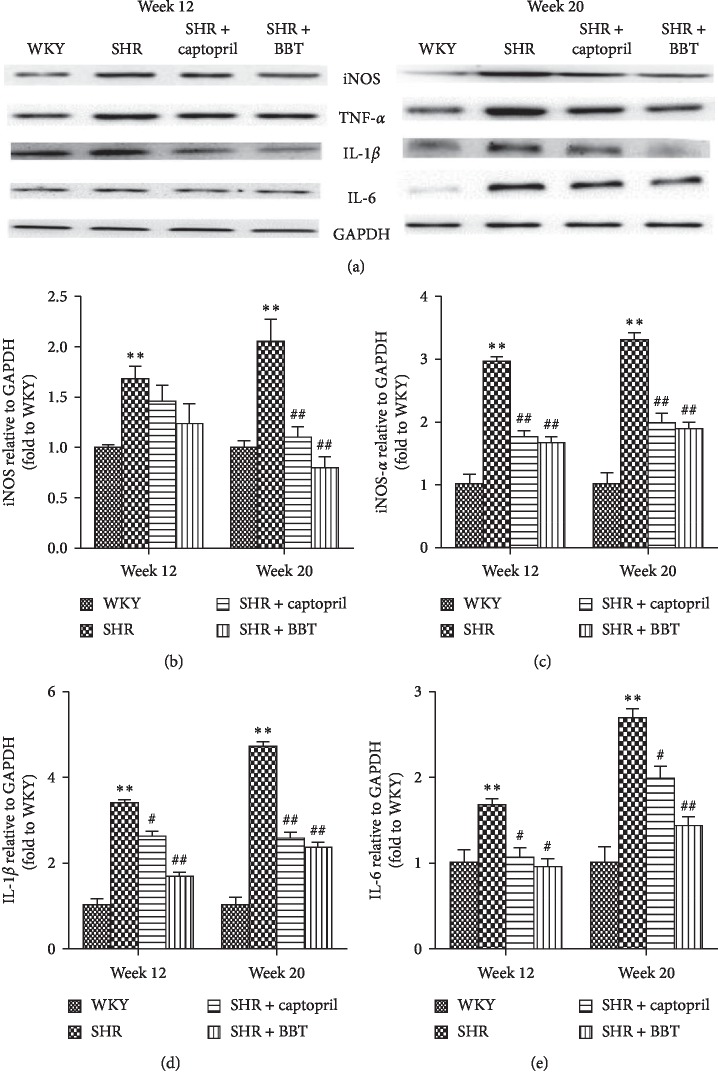
Effect of BBT on the protein expression of TNF-*α*, IL-1*β*, IL-6, and iNOS in the heart. Data were obtained by Western blot, and the results are expressed as mean ± SEM relative to GAPDH (*n* = 6 in each group). ^*∗*^*P* < 0.05 and ^*∗∗*^*P* < 0.01 vs. WKY; ^#^*P* < 0.05 and ^##^*P* < 0.01 vs. SHR.

We investigated the anti-inflammatory effects of BBT on the heart in SHRs. TNF-*α*, IL-1*β*, IL-6, and iNOS gene expression and protein expression were determined. The results for gene expression were nearly in accordance with the results for protein expression in the heart. In the SHR group, we found that the expression of TNF-*α* (*P* < 0.05), IL-1*β* (*P* < 0.05), IL-6 (*P* < 0.05), and iNOS (*P* < 0.05) were higher than that in the WKY group at 12 weeks, and the levels became even higher at 20 weeks. However, BBT significantly reduced the expressions of TNF-*α*, IL-1, IL-6, and iNOS in the heart (Figures 4 and 5).

### 3.6. Effect of BBT on NF-κB Activation

**Figure 6:**
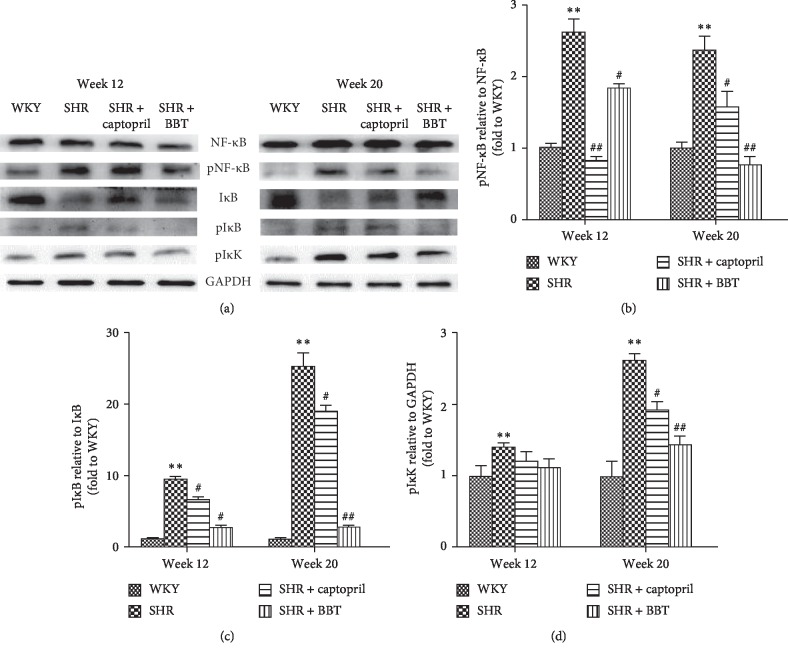
Effect of BBT on the NF-κB pathway in the heart. Data were obtained by Western blot and the results are expressed as mean ± SEM relative to GAPDH (*n* = 6 in each group). ^*∗*^*P* < 0.05 and ^*∗∗*^*P* < 0.01 vs. WKY; ^#^*P* < 0.05 and ^##^*P* < 0.01 vs. SHR.

To further explore the mechanism of inflammation in the heart, we investigated the effects of BBT on the NF-κB pathway. In the SHR group, we found that the protein expression of phospho-NF-κB–p65, phospho-IκB, and phospho-IκK was increased compared with the WKY group at week 12 in the heart and increased further at week 20. Interestingly, they were significantly reduced by BBT. Simultaneously, the expression of IκB was significantly lower at week 12 compared with the WKY group and decreased further at week 20 but was increased by BBT (Figure 6).

## 4. Discussion

BBT has been widely used as a folk formula for treating hypertension, including hypertension-induced heart damage, and the treatment is based on inflammatory processes. Thus, we investigated the anti-inflammatory effect of BBT and its related mechanism in SHR-induced heart damage. The present study showed that BBT could significantly improve the degree of heart damage in SHRs by inhibiting inflammatory processes, which was partly mediated by the NF-κB pathway.

The elementary HPLC analysis of BBT showed several chromatographic peaks demonstrating great chemical diversity. Gastrodin, guanosine, atractylenolide I, and atractylenolide III were found in BBT. They all have been reported to inhibit inflammation processes [18–20]. Thus, these chemical components could be responsible, at least in part, for the anti-inflammatory effect of BBT observed in our experiment.

First, we explored the cardiovascular protective effect of BBT in SHRs. We used common indicators to evaluate heart function. Left ventricular mass index (LVMI) was also a general marker for the detection of heart function. In our experiment, as described in Table 2, we found that levels of heart function were significantly increased at week 20 in the SHR group compared with the WKY group but that these levels could become lower after BBT treatment. Furthermore, we confirmed the protective effect of BBT on the heart using histological analyses. In accordance with the above results, BBT improved myocardial hypertrophy, according to pathological detection. Therefore, we concluded that BBT displayed a heart-protective effect in SHRs.

Furthermore, we also aimed to investigate the anti-inflammatory mechanism of BBT in the heart. The NF-κB pathway plays a key role in the occurrence and development of inflammation, including the regulation of some inflammatory mediators [21, 22]. Previous studies have demonstrated that inhibiting this transcription factor may be effective in the regulation of inflammatory processes in the heart [23, 24]. NF-κB is in an inactive state with IκB protein under normal circumstances. Once the disconnection of IκB by phosphorylation was made, in which IκK protein played a key role, NF-κB was activated [25, 26], and it participated in the immune defence response by regulating and controlling the expression of many cytokines, such as IL-1, IL-6, TNF-*α*, and iNOS, thus demonstrating a reverse induction of the NF-κB pathway [27]. In our experiment, NF-κB was activated in the heart from SHRs, and it became more serious in the process of hypertension, which is in accord with previous studies. However, we found that, in our experiment, BBT could inactive the NF-κB pathway by decreasing the level of pIκB/IκB, pNF-κB/NF-κB, and pIκK. Thus, we concluded that BBT could decrease inflammation processes by inhibiting the NF-κB pathway.

## 5. Conclusions

In conclusion, SHR-induced heart damage becomes more severe with the progression of hypertension. However, BBT may moderate this effect by inhibiting the inflammatory process, which is partly mediated by the NF-κB pathway.

## Figures and Tables

**Table 1:** Primers used for quantitative real-time PCR.

Genes	Forward primer	Reverse primer
TNF-*α*	5′-GGAGAAACCTGCCAAGTATGA-3′	5′-TACCAGGGCTTGAGCTCA-3′
IL-1	5′-CTCTCAAGCAGAGCACAG-3′	5′-TTCCATGGTGAAGTCAAC-3′
IL-6	5′-TACCCCAACTTCCAATGC-3′	5′-GATGGTCTTGGTCCTTAG-3′
iNOS	5′-ATCCCGAAACGCTACACTT-3′	5′-CGGCTGGACTTCTCACTC-3′
GAPDH	5′-GGAGAAACCTGCCAAGTATGA-3′	5′-CCCTGTTGCTGTAGCCATATT-3′

**Table 2:** Effects of BBT on LVMI. Body weight (BW), Left ventricle weight (LVW), and LVW/BW (LVMI) were measured in the heart. Data are expressed as the mean ± SEM (*n* = 6 in each group). ^*∗*^*P* < 0.05 and ^*∗∗*^*P* < 0.01 vs. WKY; ^#^*P* < 0.05 and ^##^*P* < 0.01 vs. SHR.

	WKY	SHR	SHR + captopril	SHR + BBT
*N*				
12 wks	6	6	6	6
20 wks	6	6	6	6
BW (g)				
12 wks	310 ± 20	278 ± 24	279 ± 11	275 ± 20
20 wks	327 ± 11	348 ± 8	336 ± 26	335 ± 31
LVW (g)				
12 wks	0.76 ± 0.04	0.78 ± 0.09	0.74 ± 0.06	0.82 ± 0.07
20 wks	0.66 ± 0.01	0.93 ± 0.02^*∗∗*^	0.68 ± 0.02^##^	0.81 ± 0.09^##^
LVW/BW				
12 wks	2.46 ± 0.09	2.81 ± 0.25	2.66 ± 0.28	2.99 ± 0.25
20 wks	2.03 ± 0.04	2.72 ± 0.02^*∗∗*^	2.04 ± 0.16^##^	2.41 ± 0.08^##^

## Data Availability

The data used to support the findings of this study are provided in Supplementary Materials.

## Supplementary Materials

Supplementary Materials Table 1: Effect of BBT on the blood pressure (mmHg). SHR + BBT, oral administration of BanXia BaiZhu TianMa decoction in SHRs. Data are expressed as the mean ± SEM (*n* = 6 in each group). ^*∗∗*^*P* < 0.01 vs WKY; ^#^*P* < 0.05 and ^##^*P* < 0.01 vs SHR. Table 2: Effect of BBT on the heart histology. SHR + BBT, oral administration of BanXia BaiZhu TianMa decoction in SHRs. Data are expressed as the mean ± SEM (*n* = 6 in each group). ^*∗∗*^*P* < 0.01 vs WKY; ^##^*P* < 0.01 vs SHR.
