# Supplementary material for: Heart Protection by Herb Formula BanXia BaiZhu TianMa Decoction in Spontaneously Hypertensive Rats
Source: Evid Based Complement Alternat Med. 2019 Nov 16;2019:5612929. doi: 10.1155/2019/5612929 (PMC6885217; doi:10.1155/2019/5612929)
Supplement: Supplementary Materials — Table 1: Effect of BBT on the blood pressure (mmHg). SHR + BBT, oral administration of BanXia BaiZhu TianMa decoction in SHRs. Data are expressed as the mean ± SEM (n = 6 in each group). ∗∗P < 0.01 vs WKY; #P < 0.05 and ##P < 0.01 vs SHR. Table 2: Effect of BBT on the heart histology. SHR + BBT, oral administration of BanXia BaiZhu TianMa decoction in SHRs. Data are expressed as the mean ± SEM (n = 6 in each group). ∗∗P < 0.01 vs WKY; ##P < 0.01 vs SHR. [file 5612929.f1.doc]

Table 1 Effect of BBT on the blood pressure (mmHg)

|  | 7 week | 9 week | 11 week | 13 week | 15 week | 17 week | 19 week |
| --- | --- | --- | --- | --- | --- | --- | --- |
| WKY | 136  ±3.37 | 141.75  ±5.91 | 143.75  ±2.5 | 140.75  ±3.10 | 145.25  ±2.75 | 144  ±2.16 | 142.75  ±7.14 |
| SHR | 182.75  ±6.87^**^ | 193.25  ±9.23^**^ | 209  ±10.41^**^ | 228  ±15.81^**^ | 220.5  ±18.48^**^ | 231.67  ±8.18^**^ | 237  ±6.73^**^ |
| SHR+  BBT | 183.17  ±4.40 | 196.17  ±3.76 | 204  ±5.33 | 203.33  ±10.82 | 213.5  ±8.48 | 208  ±6.03^##^ | 209.5  ±5.47^##^ |
| SHR+  Captopril | 177  ±7.07 | 157.75  ±10.90^##^ | 191  ±11.17^#^ | 192.5  ±15.24^##^ | 198.67  ±16.26^##^ | 192.66  ±5.51^##^ | 207.5  ±6.61^##^ |

***P*<0.01 *vs* WKY; ^#^*P*<0.05 and ^##^*P*<0.01 *vs* SHR

Table 2 Effect of BBT on the heart histology

|  | heart histology | |
| --- | --- | --- |
|  | 12 week | 20 week |
| WKY | 0.4±0.51 | 0.3±0.48 |
| SHR | 3.5±0.53^**^ | 4.4±0.52^**^ |
| SHR+  BBT | 2.5±0.53^##^ | 3.3±0.48^##^ |
| SHR+  Captopril | 2.7±0.67^##^ | 3±0.47^##^ |

***P*<0.01 *vs* WKY; ^##^*P*<0.01 *vs* SHR
